# Relative bioavailability, food effect, and safety of the single-dose pharmacokinetics of omecamtiv mecarbil following administration of different modified-release formulations in healthy subjects 

**DOI:** 10.5414/CP202458

**Published:** 2015-12-28

**Authors:** Rameshraja Palaparthy, Christopher Banfield, Paco Alvarez, Lucy Yan, Brian Smith, Jessica Johnson, Maria Laura Monsalvo, Fady Malik

**Affiliations:** 1 Formerly Amgen Inc.,; 2 One Amgen Center Drive, Amgen Inc., Thousand Oaks, CA, and; 3 Cytokinetics, Inc., South San Francisco, CA, USA

**Keywords:** omecamtiv mecarbil, pharmacokinetics, bioavailability, modified release, food effect

## Abstract

Objective: Omecamtiv mecarbil is a novel small molecule that directly activates cardiac myosin and increases cardiac contractility without increasing cardiac myocyte intracellular calcium. This study evaluated the relative bioavailability, food effect, and safety of several modified-release (MR) formulations of omecamtiv mecarbil. Methods: This was a phase 1, randomized, open-label, 4-way crossover, incomplete block-design study evaluating 5 MR formulations of omecamtiv mecarbil vs. an immediate-release (IR) formulation. Materials: Healthy subjects were randomized to 1 of 30 possible sequences: within each sequence, subjects were assigned to receive a single 25-mg dose of 2 of the 6 possible formulations in the fasting and/or fed states. Results: 65 subjects were screened and enrolled; 5 were replacement subjects. Pharmacokinetic and safety data were analyzed from 62 and 63 subjects in the fasting and fed states, respectively. Compared with the IR formulation, median t_max_ was longer (0.5 vs. 2 – 10 hours), and mean C_max_ was lower for all 5 MR formulations (262 vs. 34 – 78 ng/mL); t_1/2,z_ was similar (18 – 21 hours). The relative bioavailability was high (> 75%) for three MR formulations but lower (< 65%) for the other two. Overall, the effect of food on omecamtiv mecarbil pharmacokinetics was minimal for four of the MR formulations. The pharmacokinetics of the inactive metabolites M3 and M4 were similar across all formulations. Conclusions: The relative bioavailability of omecamtiv mecarbil was high (> 75%) for 3 of the five MR formulations. Food had a marginal, nonclinically meaningful effect on the pharmacokinetics of the MR formulations of omecamtiv mecarbil.

## Introduction 

Heart failure is a clinical syndrome often coupled to impaired cardiac contractility [1]. While several pharmacological and nonpharmacological interventions have been shown to improve outcomes in patients with heart failure (e.g., angiotensin-converting enzyme inhibitors, beta-blockers, aldosterone antagonists, coronary revascularization, biventricular pacing [2]), global rates of morbidity and mortality remain high [3, 4]. Inotropes, such as dobutamine, dopamine, and milrinone, increase cardiac contractility by increasing cardiac myocyte intracellular calcium, but these intracellular calcium increases have been associated with important liabilities (e.g., proarrhythmia) that limit their utility [5]. 

Omecamtiv mecarbil is a small molecule that directly activates cardiac myosin. It binds with high selectivity to cardiac myosin’s enzymatic domain, increasing the rate of adenosine triphosphatase turnover and accelerating the transition of cardiac myosin into the force-generating state [6] without affecting calcium homeostasis. Consequently, more force-generating myosin heads interact with actin filaments, increasing contractility. In preclinical models, omecamtiv mecarbil has been shown to simultaneously improve myocardial efficiency and function [7], and clinical studies have added to these results. In healthy volunteers, a 6-hour infusion of omecamtiv mecarbil at dosing rates from 0.005 to 1.00 mg/kg/h produced linear, dose-independent pharmacokinetics [8] and a maximum plasma concentration (C_max_; 9 – 1,203 ng/mL) that was proportional to the dose administered. Pharmacokinetics were similar in patients with heart failure [9]. 

The plasma protein binding of omecamtiv mecarbil in humans is ~ 82%, and it is mainly metabolized by a decarbamylation pathway, with modest metabolism, by CYP3A4 and CYP2D6. After a single dose ~ 8% of the intact parent compound can be recovered in urine collected up to 336 hours postdose, indicating extensive metabolism. Systemic clearance ranges from 132 to 207 mL/h/kg, terminal half-life from 17 to 21 hours, and apparent volumes of distribution from 3.7 to 5.2 L/kg, consistent with extensive extravascular distribution [8]. 

Omecamtiv mecarbil’s increase in cardiac contractility is tightly coupled to the resulting increase in the systolic ejection time. Evidence of intolerance can appear at plasma concentrations exceeding the maximum effects on cardiac contractility, presumably due to excessive prolongation of the systolic ejection time, which may result in myocardial ischemia [9]. 

Oral omecamtiv mecarbil is known to be highly available (> 90%) and rapidly absorbed (time to C_max_ [t_max_], 0.5 – 1.0 hours). Given that intolerance is produced by excessive omecamtiv mecarbil plasma concentrations, modified-release (MR) formulations were developed with the goal of preserving overall bioavailability while lowering C_max_ and the peak-to-trough fluctuation at steady state. This phase 1 clinical trial was designed to compare the relative bioavailability of a single 25-mg dose of five omecamtiv mecarbil MR formulations with that of the reference immediate-release (IR) matrix formulation. The effect of food on the pharmacokinetics and tolerability were also evaluated. 

## Methods 

### Design 

**Figure 1:**
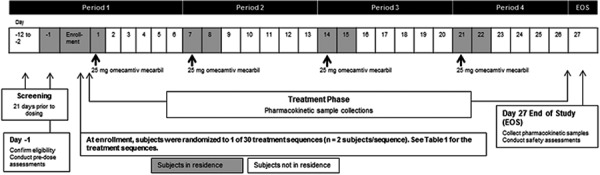
Study design. In the fasting state, subjects received omecamtiv mecarbil after an overnight fast of ≥ 10 hours; in the fed state, subjects consumed a high-fat breakfast after an overnight fast ≥ 10 hours. IR = immediate release; MP = multiparticulate capsule; MRT-F = matrix tablet; SCT-F = swellable core tablet.

This was a phase 1, randomized, open-label, 4-way crossover, incomplete block-design study conducted in healthy subjects at 1 site. Using a computer-generated randomization schedule, subjects were randomized to 1 of 30 treatment sequences, each containing 4 treatment periods; 7 days for the first 3 and 5 days for the last treatment period. Treatments were separated by a 7-day interval to allow time for wash-out, for a total study duration of ~ 28 days (Table 1) (Figure 1). Each treatment sequence was assigned to 2 subjects and included 2 of the 6 formulations administered in a fasting or fed state. The protocol and informed consent form were approved by the site’s institutional review board and all subjects provided written informed consent. Subjects who withdrew from the study before receiving omecamtiv mecarbil or for reasons other than adverse events were replaced with another subject who would receive the same randomized treatment sequence. 

### Pharmacokinetic sampling and liquid chromatography/tandem mass spectrometry (LC-MS/MS) assay 

Blood samples for the pharmacokinetic analysis were collected on day 1 predose and 0.5, 1, 2, 3, 4, 6, 8, 10, 12, 24, 48, 72, 96, and 120 hours postdose in each period using K3-EDTA collection tubes. Plasma concentrations of omecamtiv mecarbil and its metabolites M3 and M4 were extracted from 0.25 mL of human plasma aliquots using a validated solid phase extraction method and determined by LC-MS/MS with multiple reaction monitoring TurboIonSpray ionization in the positive ion mode (Worldwide Clinical Trials, Austin, TX, USA). The assay had a lower limit of quantitation of 1.00 ng/mL for omecamtiv mecarbil and 0.500 ng/mL for M3 and M4. Samples were separated on a Phenomenex, Kinetex PFP, 2.6 micron, 3.0 × 30 mm analytical column, with gradient mobile phases of ammonium acetate solution and methanol, and methanol alone. Sample concentrations were determined from weighted linear regressions of peak area ratios (peak areas of omecamtiv mecarbil, M3, M4/corresponding stable isotope labeled internal standards) vs. nominal concentrations of the calibration curve standards. Accepted runs met routine acceptance criteria of ± 15% (± 20% at the lower limits of quantitation) for accuracy of the calibration standards and quality control samples. 

### Pharmacokinetic analysis 

The plasma concentration-time data for omecamtiv mecarbil, M3, and M4 were analyzed by noncompartmental methods using WinNonlin Enterprise (version 5.1.1; Pharsight Corporation, Mountain View, CA, USA). Actual dosing and sample collection times were used in this analysis. Plasma concentrations below the lower limit of quantification of 1.0 ng/mL for omecamtiv mecarbil and 0.5 ng/mL for M3 and M4 were set to zero for the data analysis of all analytes. 

The pharmacokinetic parameters assessed were plasma concentration-time curve (area under the curve (AUC)) to time of last measureable concentration (AUC_last_), AUC to infinity (AUC_∞;_ calculated using the linear trapezoidal linear interpolation method), C_max_, t_max_, terminal elimination half-life associated with λ_z_ (t_1/2,z_), apparent plasma clearance (CL/F), the ratios of AUC_last_ of M3 and M4 to omecamtiv mecarbil, and the ratios of M3 and M4 AUC to the combined AUCs of all analytes (omecamtiv mecarbil, M3, M4 using ng×h/mL). The pharmacokinetic parameters of omecamtiv mecarbil, M3, and M4 were adjusted for each formulation by using their respective potency values where possible: IR = 0.979; MRT-F_1_ = 0.999; MRT-F_2_ = 0.995; MP = 0.984; SCT-F_1_ = 0.914; SCT-F_2_ = 0.910. 

### Safety and other assessments 

Treatment-emergent adverse events from the initial dose to the end-of-study were evaluated at the same time points within each treatment period. Adverse events were assigned to the most-recently dosed treatment period before the event occurred and were classified according to Medical Dictionary for Regulatory Activities (version 15.0). Subject incidence of adverse events was tabulated for each individual treatment and aggregated across the fasting and fed states for each formulation, and across the formulations for each condition (treatment-emergent adverse events, fatal adverse events, serious adverse events, treatment-related adverse events, treatment-related serious adverse events, and withdrawal of omecamtiv mecarbil due to adverse events). All on-study electrocardiogram (ECG) and individual vital signs, chemistry (including troponin I), hematology, and urinalysis data were assessed. 

### Statistical analyses 

To assess relative bioavailability of each MR vs. the IR formulation, natural log-transformed AUC_∞_, AUC_last_, and C_max_ were analyzed separately using a mixed-effect model, with treatment, study period, and sequence as fixed effects, and subject within each sequence as a random effect. Relative bioavailability was analyzed on pharmacokinetic data from all study periods, with the IR formulation as the reference and the MR formulations as the test treatments. To assess the effect of food on the selected MR formulation, natural log transformed AUC_∞_, AUC_last_, and C_max_ were analyzed separately using a mixed-effect model, with fasting state as the fixed effect and subject as the random effect. Statistical evaluations were conducted using SAS Software and summary statistics, adjusted parameters, and ratio values were generated using WinNonlin Enterprise (Pharsight Inc., Mountain View, CA, USA) or Microsoft Excel software. Adverse events were described using descriptive statistics. Graphs were prepared using SigmaPlot (version 11.0), (Systat Software Inc., San Jose, CA, USA). 

### Materials 

**Figure 2.**
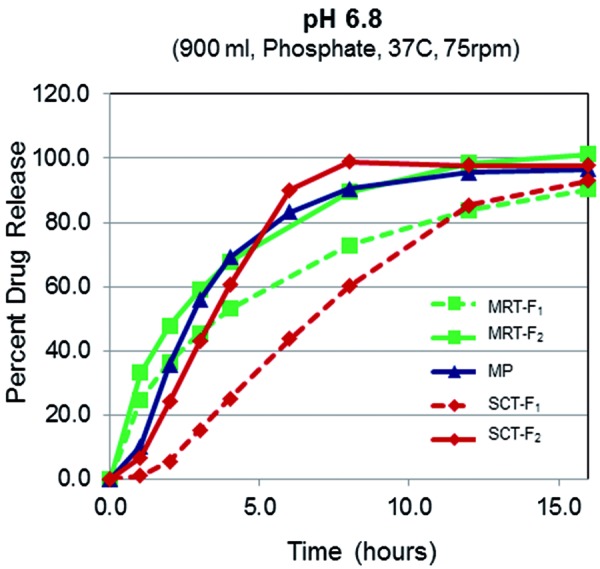
The effect of pH on the dissolution rates of 5 modified-release formulations of omecamtiv mecarbil. Normalized to 100% based on assay. MP = multiparticulate capsule; MRT-F = matrix tablet; SCT-F = swellable core tablet.

The 25-mg omecamtiv mecarbil formulations included the IR, two MR matrix tablets (MRT-F_1_, MRT-F_2_), an MR multiparticulate capsule (MP), and 2 MR swellable core tablets (SCT-F_1_, SCT-F_2_) (Table 2) (Figure 2). All two formulations were administered as a single dose containing 25 mg of omecamtiv mecarbil. 

### Subjects 

Eligible subjects were healthy males and females between 18 and 50 years with no history or evidence of clinically-relevant medical disorders, as judged by the investigator and determined by a physical examination, laboratory tests, and a 12-lead ECG. Women were without reproductive potential, and men agreed to practice highly effective methods of birth control for the duration of the study and 11 weeks after receiving the last dose of omecamtiv mecarbil. 

Exclusion criteria included a history of esophageal, gastric, or duodenal ulceration, or bowel disease; gastrointestinal surgery; or any type of malignancy within 5 years. Subjects were also excluded if they tested positive for human immunodeficiency virus, hepatitis B surface antigen, or hepatitis C antibodies; or had troponin I above the upper limit of normal at screening or on the day before dosing, or an estimated glomerular filtration rate < 80 mL/min/1.73^2^ within the screening period or on the day before dosing. Additional exclusion criteria included nicotine use; a substance abuse disorder within 1 year; treatment with agents known to affect drug metabolism, herbal medicines within 30 days, or certain over-the-counter medications; or previous exposure to omecamtiv mecarbil. Women who were breastfeeding and men with pregnant partners were also excluded. 

## Results 

### Subjects 

A total of 65 subjects received at least 1 dose of omecamtiv mecarbil; 5 were replacement subjects. A majority were white (72%) and male (85%), with a mean age of 33 years (Table 3). Six (9%) subjects discontinued the study: 2 (3%) due to an adverse event, 1 (2%) due to noncompliance, 1 (2%) due to ineligibility, and 2 (3%) due to withdrawal of full consent. The pharmacokinetics of omecamtiv mecarbil was assessed in 62 subjects in the fasting and in 63 subjects in the fed state. 

### Pharmacokinetic analysis of omecamtiv mecarbil 

**Figure 3.**
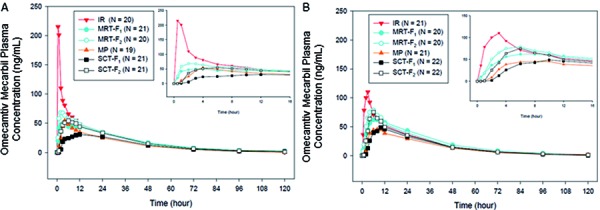
Mean plasma concentration-time profiles of 6 formulations of omecamtiv mecarbil in the (A) fasting and (B) fed states. IR = immediate release; MP = multiparticulate capsule; MRT-F = matrix tablet; SCT-F = swellable core tablet.

The mean maximum concentration was higher for the IR (C_max_ = 262 ng/mL) vs. the MR formulations, with SCT-F_1_ being the lowest (34 ng/mL) and MRT-F_2_ being the highest (78 ng/mL). Absorption was gradual for different MR formulations, with median t_max_ of 3 and 2 hours for MRT-F_1_ and MRT-F_2_, respectively; 4 hours for MP; and 10 and 6 hours for SCT-F_1_ and SCT-F_2_, respectively, compared with 0.5 hours for IR (Table 4) (Figure 3). The observed absorption was variable, yet slower (prolonged t_max_ for all MR formulations); with marginally longer median t_max_ for SCT-F_1_ and SCT-F_2_ (p = 0.046 each). The mean relative bioavailability for 3 of the MR formulations was high vs. IR: MRT-F_1_, 79%; MRT-F_2_, 87%; SCT-F_2_, 82%. The relative bioavailability was in the medium-to-low range for MP (64%) and SCT-F_1_ (58%) (Table 4). There were no notable differences in half-life between the formulations (19 – 21 hours) (Table 4). 

As with the fasting state, in the fed state, the concentrations of omecamtiv mecarbil increased gradually with lower C_max_ values for all MR formulations vs. the IR formulation (C_max_ = 144 ng/mL for IR vs. 50 – 90 ng/mL for MR) (Table 5) (Figure 3). Half-life was comparable for all MR formulations, ranging from 18 to 21 hours. 

### Food effect 

Omecamtiv mecarbil absorption was delayed in the fed state, with a longer t_max_ for IR, MRT-F_1_, MRT-F_2_, and MP formulations. For MRT-F_1_, MRT-F_2_, and MP formulations, t_max_ was doubled (3 vs. 6 hours (p = 0.0184), 2 vs. 4 hours (p = 0.0116), and 4 vs. 8 hours (p = 0.0002), respectively); there was no food effect on t_max_ for the SCT formulations (SCT-F_1_, 10 vs. 10 hours (p = 0.0522); SCT-F_2_, 6 vs. 6 hours (p = 0.3173) (Table 6). 

In the fed state, C_max_ was blunted by ~ 40% (90% confidence intervals (CI) 0.5, 0.6) and 20% (90% CI = 0.8, 0.9) for the IR and MP formulations, respectively. However, food had no effect on the total exposure (AUC_∞_) of any of the formulations, with 90% CIs contained within 80 – 125%. For MRT-F_1_, food marginally increased C_max_ (20%; 90% CI = 1.1, 1.3) and AUC_∞_ (20%; 90% CI = 1.1, 1.3). Similar marginal increases were seen in the fed state for the MRT-F_2_ and SCT-F_2_ formulations with C_max_ (< 30%; 90% CI = 1.0, 1.3 and 1.2, 1.5, respectively) and AUC_∞_ (< 20%; 90% CI = 1.0, 1.2 and 1.0, 1.1, respectively). For the SCT-F_1_ formulation in the fed state, there was an increase in C_max_ (50%; 90% CI = 1.4, 1.7) and AUC_∞_ (30%; 90% CI = 1.2, 1.4). 

### Metabolite M3 and M4 exposure 

**Figure 4.**
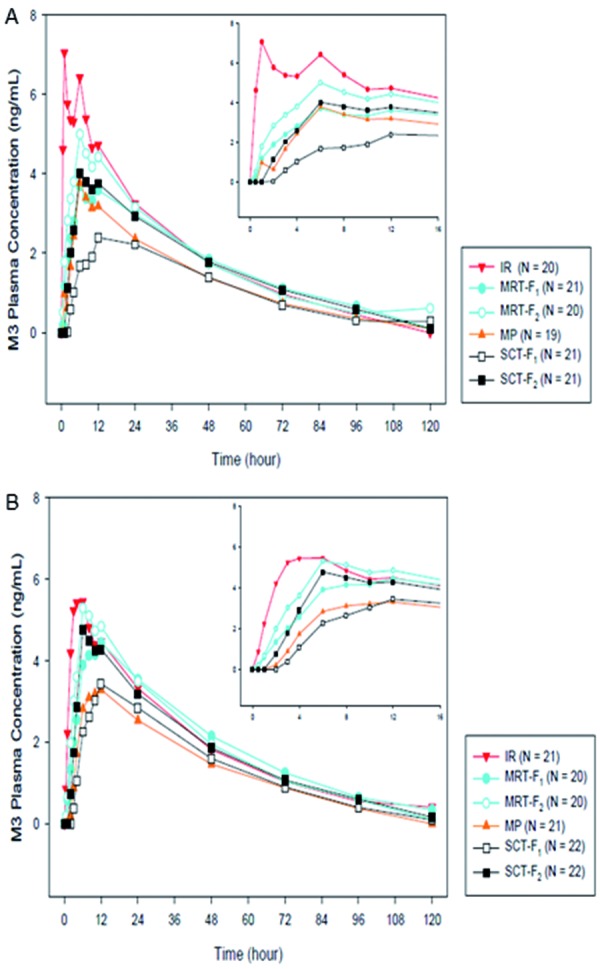
Mean M3 plasma concentration-time profiles of 6 omecamtiv mecarbil formulations in the (A) fasting and (B) fed states. IR = immediate release; MP = multiparticulate capsule; MRT-F = matrix tablet; SCT-F = swellable core tablet.

**Figure 5.**
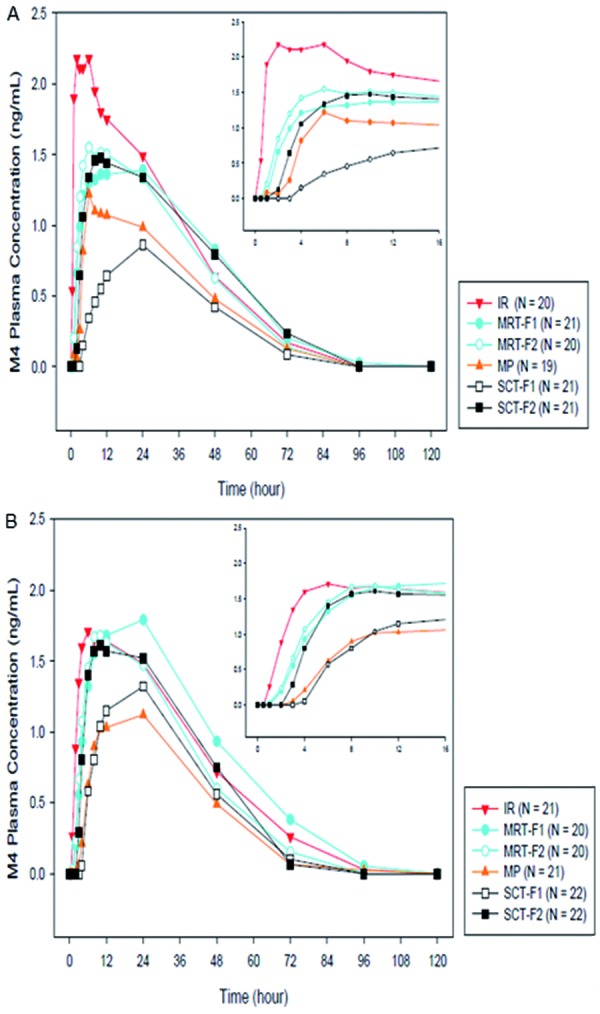
Mean M4 plasma concentration-time profiles of 6 omecamtiv mecarbil formulations in the (A) fasting and (B) fed states. IR = immediate release; MP = multiparticulate capsule; MRT-F = matrix tablet; SCT-F = swellable core tablet.

Following administration of the IR and MR formulations and similar to omecamtiv mecarbil, the concentrations of the circulating metabolites M3 and M4 gradually increased, but with a time-lag in the formation of the metabolites as confirmed by higher t_max_ values (Table 4). The mean ratio of M3 : parent was ~ 9 – 10% following administration of all 6 formulations, suggesting that formulation type did not affect total exposure of M3. Food also did not impact either peak or total exposure (Table 5) (Figure 4). Similarly, the mean ratio of M4 : parent was ~ 2 – 3% following administration of all six formulations, suggesting that formulation type did not affect the total exposure of M4, with no effect of food on peak or total exposure (Table 5) (Figure 5). 

### Safety and tolerability 

Adverse events were reported in 31% and 22% of subjects in the fasting and fed states, respectively. The most common adverse events (≥ 2 subjects) were headache (11%) and vomiting (5%) in the fasting state and headache (10%), contact dermatitis (3%), and upper respiratory tract infection (3%) in the fed state. There were no deaths or serious adverse events (Table 7). 

Two subjects discontinued the study due to an adverse event: 1 due to a urinary tract infection and 1 due to ventricular extrasystoles. In the subject with ventricular extrasystoles, the event was a telemetry finding that occurred 7 days after the last dose of the IR formulation. The subject did not experience symptoms or complaints, and 3 ECGs (2 on the same day, 1 on the 4^th^ day) did not show any changes suggestive of myocardial ischemia. The subject later had an ECG Holter, which was interpreted as normal. Troponin I at each time point was below the limit of quantification (< 0.030 ng/mL). Clinical laboratory evaluation showed no notable differences across formulations and no notable difference between the fasting and fed states. 

Six subjects with normal creatine kinase (CK) at baseline developed levels 5 – 10 times the upper limit of normal during the study. One experienced muscle trauma the day prior to the blood draw, and the CK increase was reported as a treatment-emergent adverse event; all but 1 subject had a history of physical activity. These elevations were transient, resolved spontaneously, and did not lead to study discontinuation for 5 subjects. No other laboratory values were reported as an adverse event. Three subjects had troponin I levels within the suspicious range (0.05 – 0.5 ng/mL), but below the range consistent with myocardial injury (> 0.5 ng/mL). Two occurred 6 days after drug administration, and the subjects remained asymptomatic; 1subject was coincident with a markedly elevated CK above 10 times the upper limit of normal in the context of suspected intense exercise. No clinically significant ECG changes were noted, and none of these biomarker increases were reported as treatment-emergent adverse events. 

## Discussion 

Because an increased risk of myocardial ischemia or infarction observed with high omecamtiv mecarbil concentrations is most likely due to an excessive pharmacological effect [9], 5 different MR formulations of omecamtiv mecarbil were developed with an aim of decreasing the C_max_ and peak-to-trough fluctuation, while minimally impacting total exposure. This phase 1 clinical study was designed to compare the relative bioavailability of these MR formulations with that of the reference IR formulation following administration of a single 25-mg dose and to evaluate the effect of food. 

The five formulations evaluated in this study were two matrix tablet platforms with slower (MRT-F_1_, time to release 80% of omecamtiv mecarbil (T_80_) = 10 – 12 hours) and faster release rates (MRT-F_2_ T_80_ = 6 – 8 hours), one multiparticulate capsule with a faster release rate (MP T_80_ = 4 – 6 hours), and two osmotic pump platforms with slower (SCT-F_1_, T_80_ = 8 – 12 hours) and faster release rates (SCT-F_2_ T_80_ = 4 – 6 hours). Compared with IR, none of the MR formulations showed any evidence of dose dumping, meeting one of the most important characteristics of an ideal MR formulation, with t_max_ values ranging from 2 to 10 hours. All MR formulations significantly reduced C_max_ by ≥ 70% and prolonged absorption in general. Of the MR formulations, MRT-F_1_, MRT-F_2_, and SCT-F_2_ had high relative bioavailability (> 75%); MP and SCT-F_1_ had lower relative bioavailability (< 65%). These data suggest that MRT-F_1_, MRT-F_2_, and SCT-F_2_ would achieve relatively similar total exposure as IR with lower peak exposure for a given dose. 

The food effect did not differentiate between the different MR formulations; however, in the fed state, there was a trend toward increasing AUC and C_max_ for all MR formulations except MP, which had a decrease in overall AUC and C_max_ (Table 6). Interestingly, the overall observed variability (% coefficient of variation for AUC_∞_ and C_max_) was reduced in the fed state for each of the formulations (Tables 4, Table 5). For the SCT-F_1_ formulation, food had a slightly greater effect on omecamtiv mecarbil pharmacokinetics vs. the other formulations; however, for the others the absolute change in AUC and C_max_ was < 30%. With the IR formulation, food statistically significantly reduced omecamtiv mecarbil’s C_max_, but had little effect on AUC and C_max_. The reason for this is not entirely understood, but it may be attributed to the slowing of dissolution as food tends to increase the pH of the stomach. An IR formulation is likely more susceptible to pH changes by its inherent fast-disintegrating properties, rapidly exposing the drug particles to the surrounding environment. Omecamtiv mecarbil is a biopharmaceutics classification system class 2 molecule; hence, a modifying effect of food was not unexpected, and the effect of food on omecamtiv mecarbil’s pharmacokinetics following MR formulations administration was modest and not considered clinically meaningful. However, as food appeared to reduce variability, further analyses to evaluate the value of dosing with food would be informative. 

The potency of omecamtiv mecarbil was modestly lower than the nominal intended dose of 25 mg for all formulations: in the SCT formulations, the intended potency was as low as ~ 91% due to drug substance being lost during the compression process. A secondary analysis using the potency-corrected AUC and C_max_ values showed mostly similar results and no meaningful effect on the relative bioavailability. Finally, the noncompartmental analysis-based t_1/2,z_ was similar for all formulations, with an overall mean range of 18 – 21 hours in both the fasting and fed states, consistent with earlier omecamtiv mecarbil studies [8]. 

The pharmacokinetics of the metabolites M3 and M4 were unremarkable, and the conversion of omecamtiv mecarbil to M3 and M4 was similar for the different formulations. The ratio of M3 to parent (~ 11%) and total (9%) was consistent across the formulations. The ratio of M4 to parent or total was ~ 2 – 3% and was also consistent across the formulations, indicating that metabolic activity was not dependent on the rate of input of the drug to the system. 

Single oral 25-mg doses of the MR formulations of omecamtiv mecarbil were generally well-tolerated, with no serious adverse events. In the 2 subjects who discontinued the study because of an adverse event, neither event appeared to have a causal relationship with omecamtiv mecarbil. Both events (urinary tract infection and ventricular extrasystoles) occurred 6 and 7 days after the last dose of the IR formulation, after more than 5 half-lives of the parent drug; none of the circulating metabolites had a slower elimination that could have been the probable cause of these adverse events. In the 6 subjects with elevations in CK, the disproportionate elevations over CK-MB were consistent with a skeletal muscle etiology. Elevations in CK are not uncommon in young men, the primary demographic in this study. Two additional subjects had modestly elevated asymptomatic troponin values; all 3 subjects had normal intervening cardiac troponins 24 hours following drug administration and prior to the observed elevated troponin values. 

## Conclusions 

This study evaluated the pharmacokinetics and bioavailability of omecamtiv mecarbil MR formulations relative to the IR formulation in both fasting and fed states. Compared with the IR formulation, the relative bioavailability was generally high (> 75%) for MRT-F_1_, MRT-F_2_, and SCT-F_2_, and lower for the SCT-F_1_ and MP formulations. Food had a minimal effect on the pharmacokinetics of these formulations, although it reduced overall variability. Based on these data, MRT-F_1_, MRT-F_2_, and SCT-F_2_ appear to have the desired characteristics of an MR formulation, and, as a result, were advanced for further study in patients with heart failure. 

## Acknowledgments 

The authors would like to thank Cindy Kitahara and Lindsay Henschel of Amgen Inc. for bioanalytical and sample management support; Mike Bi and Michael Kennedy of Amgen, Inc., and John Mao formerly of Cytokinetics, Inc. for formulation development work; and Janice Carlson of Amgen, Inc. for editorial support. This study was funded by Amgen Inc. 

## Conflict of interest 

This study was funded by Amgen, Inc. RP, CB, PA, LY, BS, JJ, and MLM are/were employees of Amgen. Inc. FM is an employee of Cytokinetics, Inc. 

**Table 1.** The 30 possible treatment sequences in which subjects received a single 25-mg dose of omecamtiv mercarbil.

Sequence	Period 1 (days 1 – 6)	Period 2 (days 7 – 13)	Period 3 (days 14 – 20)	Period 4 (days 21 – 26)
1	IR fasting	MRT-F_1_ fasting	MRT-F_1_ fed	IR fed
2	MRT-F_2_ fasting	IR fasting	IR fed	MRT-F_2_ fed
3	IR fasting	MP fasting	MP fed	IR fed
4	SCT-F_1 _fasting	IR fasting	IR fed	SCT-F_1 _fed
5	IR fasting	SCT-F_2 _fasting	SCT-F_2_ fed	IR fed
6	MRT-F_1_ fasting	MRT-F_2_ fasting	MRT-F_2_ fed	MRT-F_1_ fed
7	MP fasting	MRT-F_1_ fasting	MRT-F_1_ fed	MP fed
8	MRT-F_1_ fasting	SCT-F_1 _fasting	SCT-F_1 _fed	MRT-F_1_ fed
9	SCT-F_2 _fasting	MRT-F_1_ fasting	MRT-F_1_ fed	SCT-F_2 _fed
10	MRT-F_2_ fasting	MP fasting	MP fed	MRT-F_2_ fed
11	SCT-F_1 _fasting	MRT-F_2_ fasting	MRT-F_2_ fed	SCT-F_1 _fed
12	MRT-F_2_ fasting	SCT-F_2 _fasting	SCT-F_2 _fed	MRT-F_2_ fed
13	MP fasting	SCT-F_1 _fasting	SCT-F_1 _fed	MP fed
14	SCT-F_2 _fasting	MP fasting	MP fed	SCT-F_2_ fed
15	SCT-F_1 _fasting	SCT-F_2 _fasting	SCT-F_2 _fed	SCT-F_1 _fed
16	MRT-F_1_ fed	IR fed	IR fasting	MRT-F_1_ fasting
17	IR fed	MRT-F_2_ fed	MRT-F_2_ fasting	IR fasting
18	MP fed	IR fed	IR fasting	MP fasting
19	IR fed	SCT-F_1 _fed	SCT-F_1 _fasting	IR fasting
20	SCT-F_2 _fed	IR fed	IR fasting	SCT-F_2 _fasting
21	MRT-F_2_ fed	MRT-F_1_ fed	MRT-F_1_ fasting	MRT-F_2_ fasting
22	MRT-F_1_ fed	MP fed	MP fasting	MRT-F_1_ fasting
23	SCT-F_1 _fed	MRT-F_1_ fed	MRT-F_1_ fasting	SCT-F_1 _fasting
24	MRT-F_1_ fed	SCT-F_2 _fed	SCT-F_2 _fasting	MRT-F_1_ fasting
25	MP fed	MRT-F_2_ fed	MRT-F_2_ fasting	MP fasting
26	MRT-F_2_ fed	SCT-F_1 _fed	SCT-F_1 _fasting	MRT-F_2_ fasting
27	SCT-F_2 _fed	MRT-F_2_ fed	MRT-F_2_ fasting	SCT-F_2 _fasting
28	SCT-F_1 _fed	MP fed	MP fasting	SCT-F_1 _fasting
29	MP fed	SCT-F_2 _fed	SCT-F_2 _fasting	MP fasting
30	SCT-F_2 _fed	SCT-F_1 _fed	SCT-F_1 _fasting	SCT-F_2 _fasting

IR = immediate release; MP = multiparticulate; MRT-F = matrix tablet; SCT-F = swellable core tablet.

**Table 2.** Formulation characteristics.

Formulation	Description
Immediate-release (IR) tablets	Manufactured via a conventional high-shear wet-granulation process
Matrix tablets (MRT-F_1_, MRT-F_2_)	Based on hydrophilic polymer matrix systems Release rate is controlled by the polymer concentration and its molecular weight Fumaric acid is incorporated in the matrix tablet to maintain a pH-independent drug release across the intestinal pH range
Multiparticulate capsule (MP)	Formulated with coated pellets, wherein the drug is contained in the pellet core Release rate of omecamtiv mecarbil from the pellets is modulated by two successively applied functional coatings that also provide minimal pH dependence of the release across the intestinal pH range
Swellable core tablets (SCT-F_1_, SCT-F_2_)	Consist of bilayer core tablets, film-coated with an insoluble, semipermeable membrane A laser-drilled hole on the drug layer side enables osmotic-based drug release Composition is the same for both formulations Rate control is achieved by altering the ratio of insoluble polymer and water-soluble plasticizer in the semipermeable coating

**Table 3.** Baseline demographics.

Demographics	Subjects (n = 65)
Age, years	
Mean (SD)	33.0 (8.8)
Range	18 – 50
Female, n (%)	10 (15.4)
Race, n (%)	
White	47 (72.3)
Black or African American	17 (26.2)
Native Hawaiian or Pacific Islander	1 (1.5)
Hispanic or Latino ethnicity, n (%)	35 (53.8)

**Table 4.** Pharmacokinetic parameters of OM following administration of 6 formulations in the fasting state.

Pharmacokinetic parameter	IR (n = 20)	MRT-F_1_ (n = 21)	MRT-F_2_ (n = 20)	MP (n = 19)	SCT-F_1 _(n = 21)	SCT-F_2_ (n = 21)
OM						
AUC_last_ (h×ng/mL)	2,390 (385)	2,030 (432)	2,080 (623)	1,520 (284)	1,390 (489)	1,970 (436)
AUC_∞_ (h×ng/mL)	2,490 (432)	2,150 (489)	2,170 (663)	1,590 (310)	1,470 (541)	2,060 (483)
C_max_ (ng/mL)	262 (81)	60 (17)	78 (23)	61 (21)	34 (10)	62 (16)
t_max_ (h)^a^	0.5 (0.5 – 1.0)	3.0 (1.0 – 12.0)	2.0 (1.0 – 10.0)	4.0 (1.0 – 6.0)	10.0 (4.0 – 25.0)*	6.0 (2.0 – 10.0)*
CL/F (L/h)	10.3 (1.7)	12.3 (3.0)	12.5 (3.7)	16.4 (4.3)	19.2 (6.5)	12.9 (3.4)
t_1/2,z_ (h)	19.6 (4.2)	21.4 (3.4)	18.5 (4.8)	20.0 (4.4)	19.7 (4.4)	20.5 (3.8)
F_rel_ to IR (AUC_∞_)^b^	–	0.79 (0.72 – 0.86)	0.87 (0.80 – 0.95)	0.64 (0.58 – 0.70)	0.58 (0.53 – 0.63)	0.82 (0.75 – 0.90)
M3						
C_max_ (ng/mL)	7.5 (2.2)	3.9 (1.1)	5.1 (1.5)	4.7 (3.4)	2.5 (0.8)	4.2 (1.1)
AUC_last_ (h×ng/mL)	217 (53)	186 (61)	196 (61)	144 (40)	120 (55)	185 (36)
M3:OM AUC_last_ (h×ng/mL)	9.1 (1.8)	9.1 (2.3)	9.7 (2.4)	9.5 (2.1)	8.5 (2.1)	9.7 (2.0)
M4						
C_max_ (ng/mL)	2.4 (0.9)	1.6 (0.6)	1.7 (0.8)	1.4 (0.6)	1.0 (0.3)	1.6 (0.7)
AUC_last_ (h×ng/mL)	70.7 (37.8)	65.6 (35.6)	57.7 (36.6)	43.4 (28.6)	32.6 (21.4)	61.0 (35.1)
M4:OM AUC_last_ (h×ng/mL)	3.0 (1.6)	3.1 (1.4)	2.7 (1.5)	2.7 (1.8)	2.1 (1.2)	3.1 (1.6)

Data presented as mean (SD) unless otherwise specified. AUC = area under the curve; AUC_∞_ = AUC to infinity; AUC_last_ = AUC to the time of last measureable concentration; CI = confidence interval; CL/F = clearance; C_max_ = maximum plasma concentration; F_rel_ = relative bioavailability; IR = immediate release; MP = multiparticulate; MRT-F = matrix tablet; OM = omecamtiv mecarbil; SCT-F = swallowable core tablet; t_max_ = time to reach C_max_; t_1/2z_ = half-life; t_max_ = time to reach C_max_. *p < 0.046 vs. IR; analyzed using a mixed-effect model, with treatment, study period, and sequence as fixed effects, and subject with in each sequence as a random effect. ^a^median (min – max); ^b^geometric mean (90% CI).

**Table 5.** Pharmacokinetic parameters of OM following administration of 6 OM formulations in the fed state.

Pharmacokinetic parameter	IR (n = 21)	MRT-F_1_ (n = 20)	MRT-F_2_ (n = 20)	MP (n = 21)	SCT-F_1 _(n = 22)	SCT-F_2_ (n = 22)
OM						
AUC_last_ (h×ng/mL)	2,380 (433)	2,420 (390)	2,290 (520)	1,620 (440)	1,740 (492)	2,070 (329)
AUC_∞_ (h×ng/mL)	2,470 (464)	2,540 (433)	2,370 (582)	1,690 (462)	1,820 (529)	2,150 (362)
C_max_ (ng/mL)	144 (39)	70 (16)	90 (20)	50 (13)	51 (15)	79 (14)
t_max_ (h)^a^	2.0 (1.0 – 8.0)	6.0 (3.0 – 12.0)	4.0 (2.0 – 8.0)	8.0 (3.0 – 10.0)	10.0 (4.0 – 12.0)	6.0 (2.0 – 8.0)
CL/F (L/h)	10.5 (2.0)	10.1 (1.8)	11.1 (2.7)	15.9 (4.6)	14.8 (4.3)	12.0 (2.5)
t_1/2,z_ (h)	20.2 (3.6)	20.6 (3.6)	18.4 (4.6)	19.0 (5.0)	19.0 (3.9)	19.5 (3.2)
M3						
C_max_ (ng/mL)	6.7 (3.0)	4.6 (1.6)	5.7 (1.6)	3.5 (1.4)	3.5 (1.5)	5.0 (1.2)
AUC_last_ (h×ng/mL)	216 (60)	216 (66)	216 (59)	146 (52)	155 (59)	198 (42)
M3:OM AUC_last_ (h×ng/mL)	9.2 (2.6)	9.0 (2.5)	9.6 (2.1)	9.1 (2.4)	8.9 (2.1)	9.7 (1.8)
M4						
C_max_ (ng/mL)	2.0 (0.7)	1.9 (0.8)	1.9 (0.9)	1.3 (0.6)	1.4 (0.5)	1.8 (0.6)
AUC_last_ (h×ng/mL)	69.2 (38.7)	79.7 (37.7)	59.2 (39.6)	40.4 (30.2)	46.6 (27.6)	62.8 (27.6)
M4:OM AUC_last_ (h×ng/mL)	2.9 (1.6)	3.3 (1.5)	2.5 (1.6)	2.4 (1.6)	2.6 (1.3)	3.0 (1.2)

Data presented as mean (SD) unless otherwise specified. AUC = area under the curve; AUC_∞_ = AUC to infinity; AUC_last_ = AUC to the time of last measureable concentration; CL/F = clearance; C_max_ = maximum plasma concentration; IR = immediate release; MP = multiparticulate; MRT-F = matrix tablet; OM = omecamtiv mecarbil; SCT-F = swellable core tablet; t_1/2z_ = half-life; t_max_ = time to reach C_max_. ^a^median (min–max).

**Table 6.** The food effect on the pharmacokinetic parameters of omecamtiv mecarbil following administration of 6 formulations.

	IR		MRT-F_1_		MRT-F_2_		MP		SCT-F_1_		SCT-F_2_	
	Fasting (n = 20)	Fed (n = 21)	Fasting (n = 21)	Fed (n = 20)	Fasting (n = 20)	Fed (n = 20)	Fasting (n = 19)	Fed (n = 21)	Fasting (n = 21)	Fed (n = 22)	Fasting (n = 21)	Fed (n = 22)
AUC_last_ LSGM	2,372	2,351	1,855	2,256	2,097	2,343	1,492	1,571	1,331	1,719	1,950	2,094
NF/fasting (90% CI)	0.99 (0.93 – 1.05)		1.22 (1.14 – 1.29)		1.12 (1.05 – 1.19)		1.05 (0.99 – 1.12)		1.29 (1.22 – 1.37)		1.07 (1.01 – 1.14)	
AUC_∞_ LSGM	2,473	2,447	1,937	2,346	2,191	2,422	1,564	1,648	1,400	1,807	2,024	2,169
Fed/fasting (90% CI)	0.99 (0.93 – 1.05)		1.21 (1.14 – 1.29)		1.11 (1.04 – 1.17)		1.05 (0.99 – 1.12)		1.29 (1.22 – 1.37)		1.07 (1.01 – 1.14)	
C_max_ LSGM	253	139	55.1	65.5	76.8	88.9	57.8	48.3	33.1	50.1	62.3	81.1
Fed/fasting (90% CI)	0.55 (0.49 – 0.62)		1.19 (1.06 – 1.33)		1.16 (1.03 – 1.29)		0.84 (0.75 – 0.94)		1.51 (1.36 – 1.69)		1.30 (1.17 – 1.45)	
t_max_	0.5	2.0	3.0	6.0	2.0	4.0	4.0	8.0	10.0	10.0	6.0	6.0
Median p-value^a^	< 0.0001		0.0184		0.0116		0.0002		0.0522		0.3173	

AUC = area under the curve; AUC_∞_ = AUC to infinity; AUC_last_ = AUC to the time of last measureable concentration; CI = confidence interval; C_max_ = maximum plasma concentration; IR = immediate-release tablet; LSGM = least squares geometric mean; MP = multiparticulate; MRT-F = matrix tablet; NF = nonfasting; SCT-F = swallowable core tablet; t_max_ = time to reach C_max_. ^a^p value represents the difference in medians between fasting and nonfasting.

**Table 7.** Treatment-emergent adverse events.

	IR	MRT-F_1_	MRT-F_2_	MP	SCT-F_1_	SCT-F_2_
Fasting, n (%)	20	21	20	19	21	21
All adverse events	2 (25.0)	4 (19.0)	5 (25.0)	4 (21.1)	2 (9.5)	2 (9.5)
Serious adverse events	0 (0.0)	0 (0.0)	0 (0.0)	0 (0.0)	0 (0.0)	0 (0.0)
Fatal	0 (0.0)	0 (0.0)	0 (0.0)	0 (0.0)	0 (0.0)	0 (0.0)
Leading to discontinuation	1 (5.0)	0 (0.0)	0 (0.0)	0 (0.0)	0 (0.0)	0 (0.0)
Most common^a ^						
Headache	2 (10.0)	1 (4.8)	1 (5.0)	2 (10.5)	1 (4.8)	0 (0.0)
Vomiting	0 (0.0)	0 (0.0)	1 (5.0)	1 (5.3)	1 (4.8)	0 (0.0)
Nonfasting, n (%)	21	20	20	21	22	22
All adverse events	5 (23.8)	4 (20.0)	1 (5.0)	1 (4.8)	4 (18.2)	2 (9.1)
Serious adverse events	0 (0.0)	0 (0.0)	0 (0.0)	0 (0.0)	0 (0.0)	0 (0.0)
Fatal	0 (0.0)	0 (0.0)	0 (0.0)	0 (0.0)	0 (0.0)	0 (0.0)
Leading to discontinuation	0 (0.0)	0 (0.0)	0 (0.0)	1 (4.8)	0 (0.0)	0 (0.0)
Most common^a ^						
Headache	3 (14.3)	1 (5.0)	0 (0.0)	0 (0.0)	1 (4.5)	1 (4.5)
Dermatitis contact	0 (0.0)	1 (5.0)	1 (5.0)	0 (0.0)	0 (0.0)	0 (0.0)
Upper respiratory tract infection	1 (4.8)	0 (0.0)	0 (0.0)	0 (0.0)	0 (0.0)	1 (4.5)

IR = immediate release; MP = multiparticulate; MRT-F = matrix tablet; SCT-F = swellable core tablet. ^a^Occurring in ≥ 2 patients.
